# Natural disease course and genotype-phenotype correlations in Complex I deficiency caused by nuclear gene defects: what we learned from 130 cases

**DOI:** 10.1007/s10545-012-9492-z

**Published:** 2012-05-30

**Authors:** S. Koene, R. J. Rodenburg, M. S. van der Knaap, M. A. A. P. Willemsen, W. Sperl, V. Laugel, E. Ostergaard, M. Tarnopolsky, M. A. Martin, V. Nesbitt, J. Fletcher, S. Edvardson, V. Procaccio, A. Slama, L. P. W. J. van den Heuvel, J. A. M. Smeitink

**Affiliations:** 1 Nijmegen Centre for Mitochondrial Disorders, Institute for Genetic and Metabolic Disease, Radboud University Nijmegen Medical Centre, Geert Grooteplein 10, 6500 HB PO BOX 9101, Nijmegen, The Netherlands; 2 Department of Paediatrics, VU University Medical Centre, Amsterdam, the Netherlands; 3 Department of Paediatric Neurology, Radboud University Nijmegen Medical Centre, Nijmegen, The Netherlands; 4 Department of Paediatrics, Paracelsus Medical University, Salzburg, Austria; 5 Université de Strasbourg, Centre National de la Recherche Scientifique, Illkirch, France; 6 Department of Clinical Genetics 4062, Copenhagen University Hospital Rigshospitalet, Copenhagen, Denmark; 7 Department of Pediatrics, McMaster University, Hamilton, Canada; 8 Mitochondrial and neuromuscular diseases Laboratory ’12 de Octorbre’ Hospital Research Institute, Centre for Biomedical Network Research on Rare Diseases, Madrid, Spain; 9 Mitochondrial Research Group, Newcastle University, Newcastle Upon Tyne, UK; 10 Women’s and Children’s Hospital, Adelaide, Australia; 11 Pediatric Neurology Unit, Hadassah University Hospital, Jerusalem, Israel; 12 Department of Genetics, University of Angers, Angers, France; 13 Laboratoire de Biochimie, APHP-CHU de Bicêtre, Cedex, France

## Abstract

Mitochondrial complex I is the largest multi-protein enzyme complex of the oxidative phosphorylation system. Seven subunits of this complex are encoded by the mitochondrial and the remainder by the nuclear genome. We review the natural disease course and signs and symptoms of 130 patients (four new cases and 126 from literature) with mutations in nuclear genes encoding structural complex I proteins or those involved in its assembly. Complex I deficiency caused by a nuclear gene defect is usually a non-dysmorphic syndrome, characterized by severe multi-system organ involvement and a poor prognosis. Age at presentation may vary, but is generally within the first year of life. The most prevalent symptoms include hypotonia, nystagmus, respiratory abnormalities, pyramidal signs, dystonia, psychomotor retardation or regression, failure to thrive, and feeding problems. Characteristic symptoms include brainstem involvement, optic atrophy and Leigh syndrome on MRI, either or not in combination with internal organ involvement and lactic acidemia. Virtually all children ultimately develop Leigh syndrome or leukoencephalopathy. Twenty-five percent of the patients died before the age of six months, more than half before the age of two and 75 % before the age of ten years. Some patients showed recovery of certain skills or are still alive in their thirties . No clinical, biochemical, or genetic parameters indicating longer survival were found. No clear genotype-phenotype correlations were observed, however defects in some genes seem to be associated with a better or poorer prognosis, cardiomyopathy, Leigh syndrome or brainstem lesions.

**Electronic supplementary material:**

The online version of this article (doi:10.1007/s10545-012-9492-z) contains supplementary material, which is available to authorized users.

## Introduction

Mammalian complex I or NADH:ubiquinone oxidoreductase (EC 1.6.5.3.) is the largest enzyme complex of the mitochondrial oxidative phosphorylation system (Carroll et al 2006). It consists of 45 subunits, of which seven are encoded by the mitochondrial genome, and the remainder by the nuclear genome (Carroll et al 2006). Complex I has two modes of action: funnelling electrons to ubiquinone (co-enzyme Q) and redox driven proton translocation (Brandt 2006; Efremov et al 2010). These actions are carried out by three proposed functional modules, consisting of several subunits of Complex I: the N-module for NADH oxidation, the Q-module for ubiquinone reduction and the P-module for proton translocation (Angerer et al 2011). The complex has two arms, one embedded in the mitochondrial inner membrane (P-module) and one protruding into the mitochondrial matrix (N/Q-module), forming an L-shape (Clason et al 2010). The proton gradient built up by complex I-IV is used by complex V (EC 3.6.3.14) to synthesize ATP from ADP and inorganic phosphate. Fourteen highly conserved subunits can be distinguished, including the mitochondrial encoded *ND1-6, 4 L,* and *NDUFS1-3*, *NDUFS7-8* and *NDUFV1-2* (Brandt 2006). The assembly of complex I is not fully elucidated yet, but more and more assembly factors are found (McKenzie and Ryan 2010; Nouws et al 2010; Vogel et al 2007).

Mutations in one of the nuclear encoded structural or assembly genes of complex I have a dramatic effect on neurodevelopment and overall patient survival (Distelmaier et al 2009). The majority of the children with an isolated complex I deficiency (OMIM 252010) present with Leigh syndrome (OMIM 256000), a devastating neurodegenerative disease (Distelmaier et al 2009; Loeffen et al 2000; Rahman et al 1996). These patients usually present within the first months of life with psychomotor retardation in combination with signs of brainstem or extrapyramidal dysfunction and lactic acidemia (Finsterer 2008; Rahman et al 1996). The disease has first been described by Denis Leigh who described the characteristic findings on neuropathological postmortem examination with vacuolation of the neuropil and relative preservation of the neurons, associated with demyelination, gliosis and capillary proliferation (Leigh 1951), seen on MRI as bilateral hyperintensities in the basal ganglia, brainstem, thalamus, diencephalon, cerebellum and spinal cord (Rahman et al 1996). Death occurs usually within the first years of life as a consequence of respiratory failure caused by on-going brainstem dysfunction whether or not in combination with increasing muscle weakness (Finsterer 2008). Other phenotypes that have been described in patients with complex I deficiency include neonatal cardiomyopathy (Bugiani et al 2004; Distelmaier et al 2009; Hoefs et al 2008; Janssen et al 2006a, b; Saada et al 2008; Wispe et al 1992), leukoencephalopathy (Benit et al 2001; Bugiani et al 2004; Hoefs et al 2010; Zafeiriou et al 2008), fatal infantile lactic acidosis (FILA) (Bentlage et al 1996; Loeffen et al 2000) and other undefined progressive or stable encephalomyopathies (Loeffen et al 2000; Pitkanen et al 1996). The significance of the classification of symptoms into syndromes is still under debate (Distelmaier et al 2009). No obvious genotype-phenotype correlations have been identified to date and patients with mutations in the same gene may present with highly variable phenotypes (Distelmaier et al 2009; Tucker et al 2011). Also, the prognosis of nuclear encoded complex I deficiency is quite variable, ranging from fatal neonatal disease (Bentlage et al 1996; Loeffen et al 2000) to survival beyond three decades (Potluri et al 2009). For the patients and their families, it is important to get an evidence based indication of the prognosis for their child.

An accurate prognosis not only includes the age of death, but also the occurrence of symptoms such as epilepsy, visual disturbances, hearing problems, and brainstem symptoms, which may severely affect quality of life. The known presence of cardiomyopathy or deterioration during infection may have implications for follow-up and preventive immunisations. Importantly, the prediction of the clinical course of complex I deficiency is not only important for the patients and their families, it is also indispensable for establishing clinical trials. Before the effect of a drug can be tested, the natural history must be known, with the most debilitating and most prevalent symptoms identified. To date, we are not aware of any treatments or supplementation with vitamins, anti-oxidant or other compounds that positively influence the disease course of these patients (Koopman et al 2012).

In this review, we provide detailed information regarding the prognosis of patients with nuclear encoded complex I deficiency based on systematic literature search. We summarize the clinical details of all nuclear encoded complex I patients described in the literature, as well as four new cases with known mutations in complex I genes. To give a detailed overview of prevalence of clinical symptoms and their natural course in time we looked for genetic, biochemical and clinical predictors indicative for prognosis of patients and based on this provide advice to clinicians taking care of complex I deficient patients.

## Methods

### Search strategy for literature study

We searched Pubmed for all the individual subunits and assembly factors of complex I to identify cases of nuclear encoded complex I deficiency. We excluded patients with combined gene deletions. We contacted all research groups describing living patients with nuclear gene mutations and complex I deficiency presented in the literature to assess the current clinical condition and the disease course after the publication. In patients on whom limited clinical information was present, we also contacted the authors to ask for a more detailed clinical description. Siblings of patients presented in the articles on whom no biochemical or genetic analysis was performed, were excluded from this review.

### New cases

We briefly described the clinical course of four new patients with known mutations in structural or assembly genes of complex I.

### Genetic, biochemical and clinical data

We entered all data of the literature search and the new cases in a database, including age at presentation, age of death, cause of death, current age if the patient is still alive, their clinical condition, the causative gene mutation, the origin of the patient, the presence of consanguinity, and histological findings in the muscle biopsy.

The activity of complex I in skeletal muscle and skin fibroblasts was expressed as the percentage of the lowest reference value, to make the results of the measurements uniform, despite the different methods and reference values used in different laboratory. For the other patients, complex I deficiency was confirmed by other methods such as Blue Native Page. The maximum lactate, both in serum and cerebrospinal fluid (CSF) was noted and considered (arbitrary) mildly elevated above 2.1 mmol/l (noted as 1 in the supplementary table), moderately elevated above 4.0 mmol/l (2) and severely elevated above 7.0 mmol/l (3). Alanine in serum was considered increased above 450 μmol/l. The presence of tricarboxylic acid (TCA) cycle intermediates in urine was also noted.

Birth parameters were assessed, including the gestational age, birth weight and APGAR score. Low birth weight was defined as a birth weight lower than the 5th percentile for gestational age. The presence of dysmorphic features, macrocephaly, and microcephaly was noted. The age at which the following symptoms were described, was noted: psychomotor retardation, (isolated) motor retardation, developmental regression, deterioration after infection, failure to thrive, feeding problems, encephalopathy, lethargy, irritability, pyramidal signs and symptoms, extrapyramidal signs and symptoms, dys-/hypertonia, hypotonia, muscle weakness, exercise intolerance, dystrophy, ataxia, neuropathy, epilepsy, myoclonic epilepsy, ptosis, ophthalmology, nystagmus, strabismus, optic atrophy/pale optic disc, retinitis pigmentosa, vision problems, hearing loss, respiratory abnormalities, temperature regulation abnormalities, tension regulation abnormalities, dysphagia, cardiomyopathy, gastrooesophageal reflux, vomiting, constipation, hepatopathy, renal involvement, and osteoporosis. The presence and localization (basal ganglia, midbrain, brainstem, spinal cord, or cerebellum) of Leigh syndrome on brain MRI was noted, as well as the presence of cerebral/cerebellar atrophy, leukoencephalopathy, and hypoplasia of the corpus callosum. We noted the percentage of patients (literature and new cases) known to have the clinical features described above, as well as the median age at which the symptoms were first noted.

### Complex I subunits

Several sequential and parallel steps within the assembly process can be distinguished; the step in which the subunit is incorporated in the holocomplex was grouped according to Vogel et al (Vogel et al 2007). The functional modules of complex I as described in the introduction were grouped according to Angerer et al (Angerer et al 2011). Brandt analyzed whether the proteins were in the central core of the complex or accessory to it; we grouped the subunits according to his analysis (Brandt 2006).

### Statistical analysis

All data were analyzed using SPSS 16.0. Spearman’s rho was used to correlate non-parametric data. All data were described using the median and the range. The number of patients dying before a certain age was calculated as number of patients died before that age / (total number of patients who died + patients living beyond that age). Outcome variables were tested for normality if the data reflected a Gaussian distribution test. Group medians were compared using the Mann-Witney test, or the Kruskall Wallis test. Correlations were calculated using the Spearman rank coefficient. Cox survival analyses were performed for the patients with mutations in the three functional modules (Angerer et al 2011), in assembly factors compared to structural genes, as well as for patients with mutations in central subunits or accessory subunits (Brandt 2006), and in early and late in assembly (Vogel et al 2007).

Since the number of patients per gene was insufficient to perform statistical pattern analyses, we evaluated the clinical course for specific pattern in the individual genes and individual symptoms if four patients or more with mutations in these genes are known.

## Case reports

The first patient was a boy with a homozygous deletion of exons 2-4 in *NDUFAF2* c.[128-?_510 + ?del];[128-?_510 + ?del]. He was the fourth child of first cousin parents of Lebanese descent. Three older siblings were healthy. Apart from maternal diabetes mellitus, the pregnancy was uncomplicated and he was born at 36 weeks gestation by Caesarean section with normal APGAR scores. He had bilateral single palmar creases. At six months of age, he was admitted to hospital due to a Respiratory Syncytial (RS) virus infection and psychomotor retardation was noted. Nystagmus was found, which had reportedly been present since age 4 months. An ophthalmological examination showed horizontal nystagmus, hypermetropia and decreased vision. In addition he had hearing impairment and used a hearing aid, and he was found to be developmentally delayed. At the age of nine months, he was admitted because of pneumonia. He developed epilepsy with generalized seizures and was treated with Phenobarbital; EEG was normal. Respiratory support was required due to apnoeas. He had episodes of sweating and pooling of secretions was also noted. Cerebral MRI showed bilateral symmetric signal changes in putamen, substantia nigra, cerebellar peduncles and brain stem. MR spectroscopy showed elevated lactate concentration of 10 mM. Soon after admission he died. In muscle, related to CS, the activity was 0.04 (ref. range 0.20 – 0.54), and related to complex II: 0.15 (ref. range 0.43 – 1,33). Complex I activity was not measured in fibroblasts.

The second patient, with a homozygous p.Val122Met *NDUFS7* mutation, was a son of healthy, non-consanguineous Dutch parents. He had normal development until 11 months, when he regressed and his growth stagnated. The patient started vomiting and severe gastro-oesophageal reflux was found. On physical examination, nystagmus, ophthalmoplegia and hypotonia were noted. Cranial MRI showed bilateral hyperintensities in the medulla oblongata, medial thalamus and cerebral penduncles. He died at the age of 20 months, after a viral infection with rapid neurological deterioration preceding hypoventilation and coma. Complex I activity in this patient was 353 mU/U CII (reference range 783 – 1497 mU/U CII).

The third case is a boy with a homozygous p.Val122Met *NDUFS7* mutation, born from healthy, non-consanguineous Dutch parents. He developed normally until 9 months, when parents noticed he was clumsy but able to walk. At one year of age, nystagmoid eye movements were observed. At the age of 2 years and 3 months, he was admitted to a local hospital with progressive gait disturbances, lethargy and articulation problems. No laboratory abnormalities were observed. At physical examination two months later, he had a bilateral ptosis, hypertonia of all limbs, severe axial ataxia, intention tremor and hypertension. A slightly elevated lactate was found in serum and CSF fluid. MRI showed hyperintensities in the medial thalamus, brainstem and cerebellum, indicative of Leigh syndrome. One month later, he developed respiratory insufficiency, for which he was admitted to the intensive care, but continued to deteriorate. He is now 10 years old and severely disabled due to contractures and dystonia, but able to make good contact with his environment. An NADH : ubiquinone oxidoreductase activity of 24 mU/U CS (reference values 70 – 250 mU/U CS) was found in skeletal muscle.

The forth patient, with a heterozygous *NDUFV1* p.Ser56Pro and p.Thr423Met mutation, was born from healthy, non-consanguineous parents. His development was normal until the age of 8 months, when he deteriorated after an ear infection. His development regressed until the age of 11 months, followed by a partial recovery of motor skills. MRI showed white signal abnormalities, without involvement of the basal ganglia and brainstem. At present, he shows motor delay, including pyramidal signs, hypotonia and a mild ataxia, but is able to walk with support. He is 2.5 years old with minor cognitive impairment with borderline-normal language production. The NADH :ubiquinoneoxidoreductase activity was 50 mU/U CS in skeletal muscle (reference values 100 – 401 mU/U CS).

## Results

### Search results

Forty-eight articles describing patients with nuclear encoded complex I deficiency were found by searching the Pubmed database (Anderson et al 2008; Barghuti et al 2008; Benit et al 2001, 2003; Berger et al 2008; Breningstall et al 2008; Budde et al 2000, 2003; Calvo et al 2010; Dunning et al 2007; Fassone et al 2010; Fernandez-Moreira et al 2007; Ferreira et al 2011; Gerards et al 2011; Haack et al 2010; Hoefs et al 2009, 2010, 2011; Kirby et al 2004; Laugel et al 2007; Lebon et al 2007a, 2007b; Leshinsky-Silver et al 2009; Loeffen et al 2001; Loeffen et al 1998; Martin et al 2005; Mayr et al 2011; Nouws et al 2010; Ogilvie et al 2005; Ostergaard et al 2011; Pagliarini et al 2008; Pagniez-Mammeri et al 2010; Papa et al 2001; Petruzzella et al 2001; Potluri et al 2009; Procaccio and Wallace 2004; Saada et al 2008, 2009, 2011; Schuelke et al 1999; Spiegel et al 2009; Sugiana et al 2008; Triepels et al 1999; Tuppen et al 2010; van den Heuvel et al 1998; Vilain et al 2011; Zafeiriou et al 2008), identifying a total of 126 patients. We additionally describe four new complex I deficient cases of nuclearDNA origin. Ten out of 12 colleagues responded to our request for more information on patients who were still alive when the article was written or on patients who had a incomplete clinical case description.

### Genetic background

Including the new cases, the assembly factor group consists of 44 patients: two patients with a mutation in *NDUFAF1*, eight patients with a mutation in *NDUFAF2*, four patients with a mutation in *NDUFAF3*, nine patients with mutations in *NDUFAF4*, seven patients with mutations in *ACAD9*, two patients with mutations in *FOXRED1*, one patient with a mutation in *NUBPL*, eight patients with a mutation in *C20orf7*, and two patients with mutations in *C8orf38.*

The structural gene group consists of: five patients with a mutation in *NDUFA1*, one patient with a mutation in *NDUFA2*, one patient with a mutation in *NDUFA10,* five patients with a mutation in *NDUFA11*, one patient with a mutation in *NDUFA12*, ten patients with mutations in *NDUFS1*, 15 patients with a mutation in *NDUFS2*, one patient with a mutation in *NDUFS3*, 14 patients with a mutation in *NDUFS4*, six patients with a mutation in *NDUFS6*, six patients with a mutation in *NDUFS7*, three patients with a mutation in *NDUFS8*, 17 patients with a mutation in *NDUFV1* and one patient with a mutation in *NDUFV2* were found. No patients with mutations in *NDUFV3, NDUFS5*, *NDUFA3-9*, en *NDUFA12-13*, *NDUFC1-2*, *NDUFB1-11* or *NDUFAB1* were found in literature. We additionally described four patients with mutations in *NDUFAF2*, *NDUFS7*, and *NDUFV1*. Sixty children (71 %) were born from consanguineous parents. Fifty-six patients belonged to 23 families, the other patients were the only patient within the family.

### Biochemical results

Median complex I activity in muscle was 29 % of the lowest normal reference value, with a range of 3–100 % (n = 68). The three patients with normal complex I activity in muscle had a low complex I activity in fibroblasts. Complex I activity in fibroblasts had a median value of 35 % of the lowest normal reference value, with a range of 5–82 % (n = 59). Of the 14 patients reported to have abnormal findings on histology, three patients had ragged red fibres, seven had increased lipid content of the muscle, two had atrophic type 2 fibres, one had a atrophic of type 1 fibres, four had abnormal mitochondria, and two had a reduced number of type 1 fibres. In five patients, excretion of TCA cycle intermediates was reported (n = 12). Of the 96 patients in which lactate levels were reported, 86 had an increased lactate, of which 37 had an mildly increased lactate, 20 had a moderately increased lactate, and 29 had a severely increased lactate concentration. Lactate in CSF was elevated in 31 out of 34 patients of which 14 had a mild increase and 15 had a moderate increase . Serum alanine was only reported in nine patients, all but one being increased.

### Clinical details

**Fig. 1:**
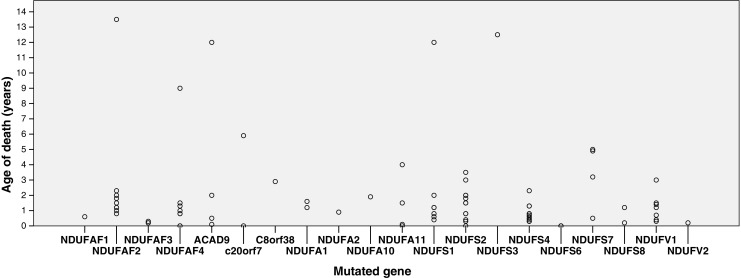
Age of death (y-axis, years) for per mutation (x-axis) of all patients in our cohort who died (n = 90)

See also Fig. 1, Table 1 and the Supplementary Table for a detailed description of the age of presentation and age of death of patients, categorized by gene mutation, including the new cases described. Sixty-seven boys (58 %) and 49 girls were reported; of 15 patients no gender was mentioned in the case report. The median age at presentation was four months (range 0 months to nine years; n = 130). Median age of death was 10 months (range 0 months to 13.5 years; n = 90). Thirty-four patients died before the age of six months (25 %), 47 died before the age of one year (36 %), 74 patients died before the age of two years (58 %), 81 patients died before the age of four years (66 %) and 85 patients died before the age of ten years (75 %). The patients who are still alive are now six months to 38 years old (median age 9 years; n = 33). Nine patients were reported to have died from cardiorespiratory failure, 15 patients died from respiratory failure, six patients died from central hypoventilation, three patients died from multiple organ failure, 21 patients died from lactic acidosis, two patients suffered from aspiration pneumonia, three patients from cardiomyopathy, and four patients died from infection.

**Table 1:** Age of death (range, in years) per mutation; > means beyond the age of

Gene mutated	Range age of death (years)	Number of observations
NDUFAF1	0.6 – >24	2
NDUFAF2	0.8 – 13.5	9
NDUFAF3	0.2 – 0.3	4
NDUFAF4	0 – 1,5	9
ACAD9	0.1 – >12	7
FOXRED1	>10 - > 22	2
NUBPL	>8	1
C20orf7	0 – >29	8
C8orf38	> 1,8 – 2.9	2
NDUFA1	1.2 – >38	5
NDUFA2	0.9	1
NDUFA10	1.9	1
NDUFA11	0 – 4	5
NDUFA12	> 10	1
NDUFS1	0.4 – >4	10
NDUFS2	0.3 – >17	15
NDUFS3	13	1
NDUFS4	0.3 – 2,3	14
NDUFS6	0	6
NDUFS7	0.5 – 5	6
NDUFS8	0.2 – >9	3
NDUFV1	0.3 – >13	17
NDUFV2	0.2	1

In seven patients, a microcephaly was described, one patient had macrocephaly with wide anterior fontanel, in one patient a hydroureter with hydronephrosis and in one child upslanting palpebral fissures and hypospadias were reported. The latter patient was a child of consanguineous parents. For a more detailed summary of the prevalence and age of presentation of the individual symptoms, see Table 2.

**Table 2:** Reported clinical symptoms in patients with nuclear encoded complex I deficiency, including the prevalence and mean age of presentation in our cohort (n = 130)

Clinical symptom	Prevalence	Median age of onset (months)	Range (years)
Hypotonia	60 %	5	0 – 6
Failure to thrive	34 %	6	0 – 2.8
Nystagmus	34 %	7	0 – 10
Dys- or hypertonia	32 %	12	0 – 9
Psychomotor retardation	30 %	6	0 – 6
Feeding problems	29 %	5	0 – 5.2
Pyramidal symptoms	28 %	13	0 – 13
Respiratory abnormalities	27 %	11	0 – 12
Developmental regression	25 %	11	0 – 7,5
Vomiting	22 %	7	0 – 4
Epilepsy	21 %	8	0 – 10
Cardiomyopathy	20 %	4	0 – 3
Optic atrophy	20 %	10	0 – 11
Deterioration after infections	18 %	6	0.3 – 3.2
Ataxia	18 %	24	0.5 – 8
Lethargy	18 %	6	0 – 20
Encephalopathy	16 %	6	0 – 2.5
Vision problems	17 %	6	0 – 10
Muscle weakness	15 %	8	0 – 11.6
Irritability	15 %	7	0 – 16
Extrapyramidal symptoms	15 %	14	0.3 – 10
Strabismus	14 %	7	0.1 – 6
Dysphagia	13 %	6	0 – 9
Pure motor retardation	11 %	9	0 – 2
Dystrophy	9 %	6	0.3 – 8
Myoclonic epilepsy	8 %	10	0.2 – 10
Hearing loss	8 %	16	0.1 – 10
Ptosis	7 %	16	0.3 – 2.5
Exercise intolerance	7 %	48	0 – 20
Temperature regulation problems	5 %	6	0.4 – 20
Gastrooesophageal reflux	5 %	6	0 – 1,1
Hepatopathy	4 %	4	0 – 1,3
Ophthalmoplegia	4 %	9	0.6 – 2.2
Constipation	4 %	6	0.2 – 7
Osteoporosis	3 %	78	5-16
Neuropathy	3 %	8	0.5 – 30
Tension abnormalities	2 %	51	0.5 – 8
Renal involvement	2 %	17	0.5 – 2
Retinitis pigmentosa	1 %		13

Sixty-five patients with Leigh syndrome were described, of which 47 had hyperintensities in the basal ganglia and thalamus, 14 had lesions in the midbrain, and 23 had lesions in the brainstem. Spinal cord hyperintensities were described in one patient and lesions in the cerebellum were reported in four patients. Fourteen patients had cerebral atrophy and six patients had cerebellar atrophy. Leukoencephalopathy was present in 26 patients. Hypoplasia of the corpus callosum was present in three patients. In two patients, no abnormalities on brain MRI were described. For a detailed description of the MRI abnormalities in our patients, see Table 3. Of the 50 patients who had lesions in the basal ganglia or midbrain, 27 had pyramidal or extrapyramidal features (54 %). Of the 23 patients with hyperintensities in the brainstem, 20 had features of brainstem dysfunction (87 %) and 13 had pyramidal or extrapyramidal signs (56 %). Of the 24 patients with leukoencephalopathy, 17 had pyramidal or extrapyramidal signs (70 %).

**Table 3:** The prevalence of MRI abnormalities in patients with nuclear encoded complex I deficiency (n = 91)

MRI abnormality	Prevalence
Leigh syndrome	84 %
basal ganglia	53 %
midbrain	17 %
brainstem	27 %
spinal cord	1 %
cerebellum	4 %
Cerebral atrophy	12 %
Cerebellar atropy	9 %
Leukencephalopathy	28 %
Hypoplasia of the corpus callosum	3 %
Normal MRI	2 %

### Statistical analysis

**Fig. 2:**
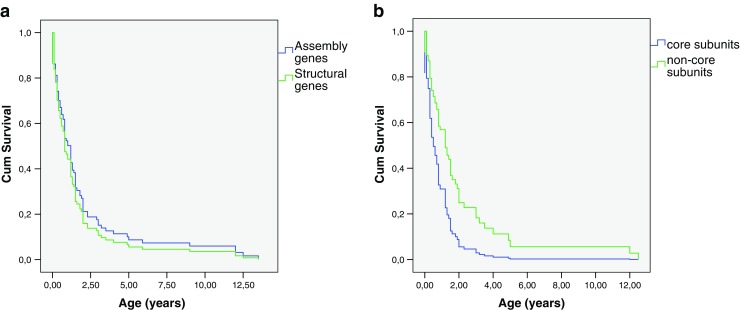
Cox regression survival curve of complex patients. Age in years (x-axis) and cumulative survival (y-axis). **a** Survival of patients with mutations in assembly genes (blue) compared to patients with mutations in structural genes (green) (b = -0.169; p = 0.457; n = 90). B: Survival of patients with mutations in core subunits (blue) compared to patients with mutations in non-core genes (green) (b = 0.732; p = 0.007; n = 61)

No correlation between complex I activity and age of onset (p = 0.377; n = 82) or age of death (p = 0.145; n = 53) was found. A moderate correlation was found between age of presentation and age of death (σ = 0.582; p = <0.001; n = 91). No significant differences in age of death or age of onset were found between the functional subunits, order within the assembly of the holo-complex, or the sub-complex in which the protein is present. Survival of patients with mutations in assembly genes did not differ from patients with mutations in structural genes (p = 0.457). Survival of patients with mutations in core subunits was lower than of patients with mutations in non-core genes (b = 0.732; p = 0.007; n = 61). Survival of patients with mutations in genes encoding proteins built in early in assembly did not differ from survival of patients with mutations in genes encoding subunits which were built in late in the assembly (p = 0.513). No difference could be observed between patients having mutations in different functional subunits (p = 0.775). See also Fig. 2.

## Discussion

In this review, we describe the disease course of 130 patients with mutations in either structural or assembly proteins leading to complex I deficiency. The disease course of nuclear encoded complex I deficiency is quite homogeneous, with ultimately most children having a severe multi-system disease with prominent neurological involvement. Most children with an age of younger than six months presented with hypotonia, feeding problems and failure to thrive, vomiting, encephalopathy, epilepsy, and eye movement disturbances. Children beyond the age of six months more frequently presented with psychomotor retardation or developmental regression, pyramidal signs and symptoms, dystonia, ataxia, epilepsy, failure to thrive, vomiting, and optic atrophy. The most specific signs pointing to a nuclear encoded complex I deficiency are brainstem involvement, optic atrophy and Leigh syndrome characteristics on MRI. Cardiac, renal, or hepatic involvement was seen in 24 % of the children. Deterioration with infections was described in only 18 % of the patients, which is less frequent than generally found in children with mitochondrial disorders and may therefore be an underreporting. No dysmorphic features were present, except for two children of consanguineous parents. Most children appear normal at birth, five percent of the patients were born mildly premature and seven percent had a low birth weight, similar to what was previously described (Distelmaier et al 2009). Increased lactic acid concentration was present in 90 % of the patients.

Importantly, not all patients with a nuclear encoded complex I deficiency have a poor prognosis and quality of life. For example the girl with a compound heterozygous *NDUFV1* mutation described by Zafeiriou et al (Zafeiriou et al 2008) has developed normally after the initial regression around the age of one year. She has learning problems and mild spasticity, but is able to ride a bicycle and walk without support. The patient with a compound heterozygous mutation in NDUFAF1, described by Dunning et al (Dunning et al 2007), is still alive at the age of 24 years. He works part time, he has Asperger syndrome and kyphosis, but has a sense of humor and is generally cheerful.

Only two patients without abnormalities on MRI were reported (Saada et al 2009). Possibly, these siblings would have developed brain abnormalities if they had lived longer than three months. Of the patients dying before the age of six months, two patients were reported without any brain abnormalities (15 %), whereas only four patients older than six months (6 %) were described with brain atrophy without Leigh syndrome or leukoencephalopathy. No clear correlation between the anatomic location of the hyperintensities and the clinical symptoms was observed.

Historically, no obvious mutation-related phenotype apparent in complex I deficiency was reported (Distelmaier et al 2009). For example, cardiomyopathy has first been reported as characteristic for the *NDUFS2* gene (Loeffen et al 2001) but was later reported in other nuclear complex I genes as well (Distelmaier et al 2009). Moreover, after the first three patients with *NDUFS2* mutations with cardiomyopathy, no patients with this combination have been described (Supplementary table) (Tuppen et al 2010). Besides, mutations in the same gene can express a high variety of clinical and biochemical phenotypes, even within the same family (Budde et al 2003). If more than four patients with mutations in a certain gene have been described we analyzed the genotype-phenotype relation. We found that cardiomyopathy was more prevalent in patients with *ACAD9* and *NDFUA11* mutations. In patients with *NDUFAF2* mutations, brainstem symptoms and Leigh syndrome on MRI were observed in all patients, as well as a high prevalence of pale optic discs or optic atrophy. Patients with *NDUFAF4* and *NDUFS6* patients seem to have a very poor prognosis, but since all *NDUFAF4* patients are from the same family, no solid conclusions can be drawn. In patients with *NDUFA1* mutations, hearing loss seems more common and prognosis is often quite good. Leukoencephalopathy seems more prevalent in patients with *NDUFS1* and *NDUFV1* mutations, but mutations in both genes may also lead to Leigh syndrome. Patients with *NDUFS4* and *NDUFS7* mutations almost invariably have Leigh syndrome, in patients with *NDUFS7* mutations, the brainstem is often affected.

The prognosis of nuclear complex I deficiency is generally very poor (Figs. 1 and 2), however quite a few exceptions exist. More than half of the patients died before the age of two years and 79 % died before the age of ten years. In comparison, children with a mitochondrial DNA encoded complex I deficiency present at the median age of 12 months and have a tendency toward a longer survival than patients with nuclear encoded complex I deficiency (Swalwell et al 2011). Although lactic acidosis was described as the cause of death in 41 % of the patients dying before the age of six months, a high lactate is not necessarily associated with a poor prognosis. The same applies to a high lactate in the CSF. No correlation between the age of death and the location of the protein within the assembly, function or structure of complex I could be elucidated. Strikingly, within one family harbouring the same mutation, one child may die before the age of one year whereas a sibling lives into the first decade (Saada et al 2008). In the patients living into their first decade, no lower prevalence of cardiomyopathy, brainstem dysfunction, Leigh syndrome or a higher complex I activity could be observed. Although patients with mutations in core subunits have a significantly poorer prognosis than patients with mutations in non-core subunits, the variability in prognosis is extremely high. The value of predicting outcome based on this kind of molecular characteristics warrants further research, e.g., *in silico*.

In previous studies in a heterogeneous group of children with mitochondrial disorders, cardiomyopathy was found to be a significant predictor of a fatal outcome (Scaglia et al 2004). However, since the neurological phenotype of patients with nuclear encoded complex I deficiency is so overwhelming, this observation is difficult to confirm in our cohort. For example, all patients with mutations in complex I assembly genes and cardiomyopathy survived beyond the age of five years, although all children with mutations in structural genes and cardiomyopathy died before the age of two years.

Cardiomyopathy may be present in both patients with structural and assembly factor mutations, and in combination with various clinical phenotypes ranging from lactic acidemia and progressive encephalomyopathy (Loeffen et al 2001), to mild and stable neurological syndromes (Dunning et al 2007; Haack et al 2010). Although cardiomyopathy always presented before the age of three years in this cohort, cardiac function should be checked regularly in all patients with nuclear encoded complex I deficiency. We also advice to regularly check the ocular manifestations of the disease, for it is important to adapt the environment to visual disturbances in children with optic neuropathy, severe ptosis or ophthalmoplegia (McFarland et al 2010). General advices, such as tube-feeding in case of malnutrition or aspiration, and aggressively treating fever and infectious diseases also apply to these patients to prevent secondary malfunction or challenging of the energy metabolism (Morava et al 2006). For patients with dystonia or spasticity, a combination of physiotherapy and pharmacotherapy may relieve symptoms (McFarland et al 2010).

This is the first retrospective study of the clinical disease course of patients with a nuclear encoded complex I deficiency. Although the number of patients analyzed is high considering the prevalence of this condition, the number of patients with particular gene mutations is still too low to see clear genotype-phenotype patterns. Besides, an obvious report bias exists and the quality of our data is inherently limited by the quality and completeness of the data reported by other research groups. Some case reports were very brief and only reported the main symptoms. Furthermore, it is more likely that the symptoms present in previously reported patients with the same mutation will be diagnosed and reported, e.g., optic atrophy or retinitis pigmentosa. The median and mean age we calculated was based on the observation and description of others, sometimes in only a few papers. Therefore, the numbers in our paper should be carefully interpreted and interpreted in a clinical context.

For future research, we would suggest to perform prospective follow-up of these patients, for example with the Newcastle Paediatric Mitochondrial Disease Scale (Phoenix et al 2006). Only a prospective follow-up will provide valid data to be used in the preparation clinical trials in predicting the natural disease course or selecting relevant outcome measures.

We also suggest to continue publishing clinical details of these rare diseases. Only more case descriptions will enable us to predict the natural disease course of patients with these rare diseases, which is not only useful for future clinical trials, but is also indispensible for the patients and their families.

## Conclusion

In conclusion, nuclear encoded complex I deficiency generally has a devastating clinical disease course, characterized by a severe neurological phenotype including brainstem involvement and optic atrophy, in combination with lactic acidosis and Leigh syndrome on MRI. Most patients die before the age of one year, but patients in their third decade have been described. We advise to regularly check cardiac and ocular manifestations of the disease, to optimize nutrition, to treat intercurrent illnesses promptly, and to provide adequate symptomatic relief and family support.

## Electronic supplementary material

Below is the link to the electronic supplementary material.

ESM Supplementary Table 1 Genetic, biochemical and clinical features of patients with nuclear encoded complex I deficiency. Cause of death: Respiratory failure (RF), Cardiorespiratory failure (CRF), Central Hypoventilation (CH), Lactic acidosis (LA), Cardiomyopathy (CM), Brainstem dysfunction (BD), Infection (INF), Multi Organ Failure (MOF), Aspiration Pneumonia (AP), Sudden death (SD); Lactate serum and CSF: mildly increased (1): 2.1 – 4 mmol/l; moderately increased(2) 4.0 – 7.0 mmol/l; severely increased (3): > 7.0 mmol/l; Muscle biopsy: ragged red fibers (RRF), atrophy type 2 fibers (AT2F), lipid accumulation(LPA), atrophic fibers (AF), atypical mitochondria (AM), reduced number of type 1 fibers (RNT1F). (XLS 143 kb)
